# Unexpected multiplicity of QRFP receptors in early vertebrate evolution

**DOI:** 10.3389/fnins.2014.00337

**Published:** 2014-10-24

**Authors:** Dan Larhammar, Bo Xu, Christina A. Bergqvist

**Affiliations:** Unit of Pharmacology, Science for Life Laboratory, Department of Neuroscience, Uppsala UniversityUppsala, Sweden

**Keywords:** QRFP, QRFP receptor, RFamide peptide, 26RFamide, G protein-coupled receptor, coelacanth, Latimeria chalumnae, spotted gar

## Abstract

The neuropeptide QRFP, also called 26RFa, and its G protein-coupled receptor GPR103 have been identified in all vertebrates investigated. In mammals, this peptide-receptor pair has been found to have several effects including stimulation of appetite. Recently, we reported that a QRFP peptide is present in amphioxus, *Branchiostoma floridae*, and we also identified a QRFP receptor (QRFPR) that mediates a functional response to sub-nanomolar concentrations of the amphioxus peptide as well as short and long human QRFP (Xu et al., submitted). Because the ancestral vertebrate underwent two tetraploidizations, it might be expected that duplicates of the QRFP gene and its receptor gene may exist. Indeed, we report here the identification of multiple vertebrate QRFPR genes. Three QRFPR genes are present in the coelacanth *Latimeria chalumnae*, representing an early diverging sarcopterygian lineage. Three QRFPR genes are present in the basal actinopterygian fish, the spotted gar. Phylogenetic and chromosomal analyses show that only two of these receptor genes are orthologous between the two species, thus demonstrating a total of four distinct vertebrate genes. Three of the QRFPR genes resulted from the early vertebrate tetraploidizations and were copied along with syntenic neuropeptide Y receptor genes. The fourth QRFPR gene may be an even older and distinct lineage. Because mammals and birds have only a single QRFPR gene, this means that three genes have been lost in these lineages, and at least one of these was lost independently in mammals and birds because it is still present in a turtle. In conclusion, these results show that the QRFP system gained considerable complexity in the early stages of vertebrate evolution and still maintains much of this in some lineages, and that it has been secondarily reduced in mammals.

## Introduction

Many of the biologically active peptides that end with the sequence RFamide, i.e., arginine-phenylalanine-amide, bind to receptors that are more closely related to each other than to other G protein-coupled receptors. These peptides include NPFF, GnIH, PRLH, and QRFP/26RFa, henceforth called QRFP (Fukusumi et al., 2006; Osugi et al., 2006, 2011; Elphick and Mirabeau, 2014). Furthermore, peptides ending with RYamide, namely the neuropeptide Y (NPY) family including peptide YY (PYY) and pancreatic polypeptide (PP), bind to a large family of receptors closely related to those responding to the peptides listed above (Sundstrom et al., 2008). Thus, many RFamide/RYamide peptides have receptors that form an evolutionary clade. On the other hand, some peptides with carboxyterminal RFamides bind to more distantly receptors, indicating that some peptides have found their receptor partners in independent evolutionary events, such as kisspeptin.

The NPY receptor family is probably the largest peptide GPCR family in vertebrate genomes with seven ancestral members (Larsson et al., 2008, 2009; Larhammar and Bergqvist, 2013) and with five members present in many presently living mammals. This family consisted of three ancestral linked genes before the origin of vertebrates, then this triplet was quadrupled (Larhammar and Salaneck, 2004) in the two ancestral vertebrate tetraploidizations called 1R and 2R (Nakatani et al., 2007; Putnam et al., 2008). After 2R, gene losses reduced the number to a total of 7 NPY-family receptors in the ancestor of gnathosomes, i.e., the jawed vertebrates (Larsson et al., 2009; Larhammar and Bergqvist, 2013). The RFamide peptides, in contrast, have quite modest receptor repertoires in mammals with just a single receptor each for the four peptides NPFF, GnIH (called RFRP-3 in mammals), PRLH and QRFP, albeit NPFF and GnIH can activate also one another's receptors (Liu et al., 2001; Mollereau et al., 2002). This might be taken as evidence that these four receptors arose as a result of the two tetraploidizations. Recently, however, the PRLH receptor family alone was found to consist of as many as four members in a broader vertebrate perspective, probably as a result of the two basal vertebrate tetraploidizations (Kuraku and Kuratani, 2011). Furthermore, we have recently identified and characterized a QRFP receptor and its peptide ligand from amphioxus (Xu et al., submitted), implying that all four RFamide peptides were already established before the vertebrate tetraploidizations. The origin of the four RFamide peptides and their receptors before the emergence of vertebrates is also supported by global analyses of genomes for the presence of peptide and receptor genes (Jekely, 2013; Mirabeau and Joly, 2013; Elphick and Mirabeau, 2014).

Our searches in the ENSEMBL genome databases retrieved multiple QRFPR-like sequences from many vertebrate species and our phylogenetic analyses showed that these sequences represent clades with deep roots in the vertebrate tree. We therefore proceeded to complement the sequence-based phylogenetic analyses with information about the chromosomal locations of the QRFPR genes in the genomes with most reliable assemblies in order to obtain clues to the mechanisms of gene duplication. This would allow more precise determination of the time points for the gene duplications. Our studies build upon our previous extensive analyses of the NPY receptor family genes, some of which are located on the same chromosome as the QRFPR gene in the human genome. In addition, we have incorporated information from the recently completed genome of a basal ray-finned fish, the spotted gar, *Lepisosteus oculatus*, which in many instances has been found to have retained a large proportion of the ancestrally duplicated vertebrate genes, and to have undergone comparatively few chromosomal rearrangements (Amores et al., 2011). Furthermore, this species diverged before the teleost ancestor underwent its third tetraploidization, 3R (Jaillon et al., 2004), making it simpler to analyze than many teleost fishes. Another useful species for this type of comparison thanks to its slow evolution is the African coelacanth, *Latimeria chalumnae*, which has many examples of retained ancestral genes, albeit its genome assembly does not have contigs that are sufficiently large to allow comparisons of conserved synteny except in a few cases (Amemiya et al., 2013).

In contrast to a recent study that proposed three ancestral vertebrate QRFPR subtypes (Ukena et al., 2014), our analyses identified four ancestral vertebrate QRFP receptor genes. We present here the evidence for this conclusion and propose a likely scenario for the gene duplications in early vertebrate evolution in relation to the vertebrate tetraploidizations. These findings imply that mammals have lost much of the QRFP system, possibly explaining why the single QRFP peptide found in mammalian genomes seems to have diverged from other vertebrates which still retain more extensive QRFP receptor repertoires.

## Materials and methods

**Figure 1:**
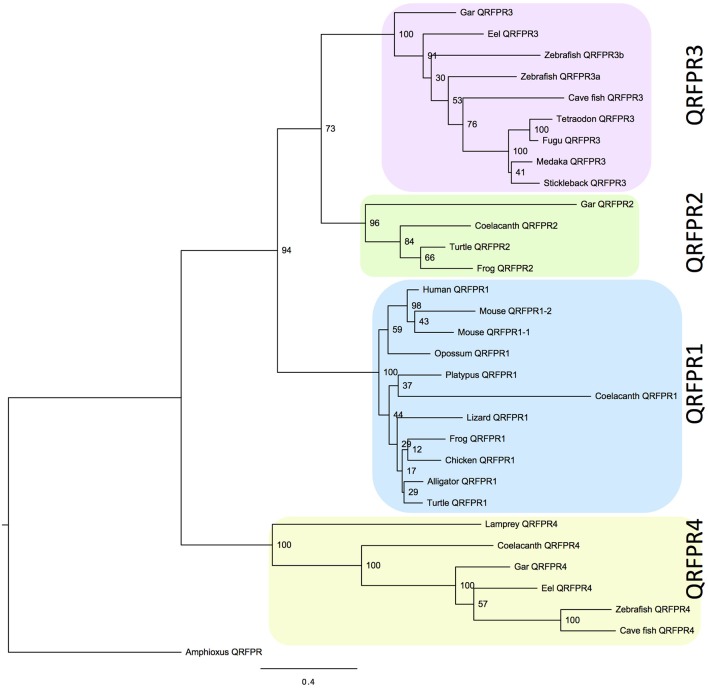
**Phylogenetic tree calculated with the maximum likelihood (ML) method showing the relationships of the QRFPR receptors for human (*Homo sapiens*), mouse (*Mus musculus*), gray short-tailed opossum (*Monodelphis domestica*), platypus (*Ornithorhynchus anatinus*), chicken (*Gallus gallus*), American alligator (*Alligator mississippiensis*), anole lizard (*Anolis carolinensis*), Chinese soft-shelled turtle (*Pelodiscus sinensis*), western clawed frog (*Xenopus tropicalis*), African coelacanth (*Latimeria chalumnae*), spotted gar (*Lepisosteus oculatus*), Japanese eel (*Anguilla japonica*), zebrafish (*Danio rerio*), cave fish (*Astyanax mexicanus*), medaka (*Oryzias latipes*), three-spined stickleback (*Gasterosteus aculeatus*), fugu (*Takifugu rubripes*), green spotted pufferfish (*Tetraodon nigroviridis*), sea lamprey (*Petromyzon marinus*)**. The amphioxus QRFPR (*Branchiostoma floridae*) was used as out-group to root the tree (Xu et al., submitted). The tree was calculated using PhyML3.0 (Guindon et al., 2010) and the complete sequences aligned with MUSCLE. For alignment see Supplementary Figure 1.

The Ensemble sequence database was searched with Blast/Blat using as query sequence the human QRFP receptor. As new receptor sequences were found and confirmed to be QRFPR-related, they were used as queries in additional searches. A list of the genes whose predicted receptor sequences were analyzed phylogenetically is shown in Supplementary Table 1 which contains their accession numbers. Amino acid alignments were made in Jalview 2.8 version 14.0 (Waterhouse et al., 2009) using the MUSCLE web tool with standard settings. During the course of the work, phylogenetic Neighbor-Joining (NJ) (Saitou and Nei, 1987) trees were made by using Clustal X version 2.0.12 (Larkin et al., 2007), with standard settings and 1000 bootstrap replicates. The tree shown in Figure 1 was made with the Maximum Likelihood (ML) method using the PhyML3.0 algorithm (Guindon et al., 2010) with the following settings: amino acid frequencies (equilibrium frequencies), proportion of invariable sites (with optimized p-invar) and gamma shape parameters were estimated from the alignments, the number of substitution rate categories was set to 8, BIONJ was chosen to create the starting tree, both NNI and SPR tree optimization methods were considered and both tree topology and branch length optimization were chosen.

## Results

### Phylogenetic analyses of QRFPR sequences

Extensive searches in vertebrate genomes identified three QRFPR genes in three species, namely in spotted gar, coelacanth, and zebrafish. Several other genomes were found to contain two genes, these include the western clawed frog *Xenopus tropicalis*, the Chinese soft-shelled turtle, the Japanese eel, and the cave fish. Two genes were also found in the anole lizard and the American alligator, but in these species one of the genes was incomplete and could not be included in the phylogenetic analysis. Finally, it has been known since before that mouse as well as rat has two receptor genes as a result of a recent gene duplication (Takayasu et al., 2006), probably in the rodent lineage.

As the QRFP gene has five introns in the vertebrate genes, several annotations contained obviously erroneous predictions of splice junctions. Also, the beginning and end of some genes were missing. After manual curation of the sequences to comply with consensus rules, the sequences were aligned with MUSCLE in Jalview (see Materials and Methods) and analyzed phylogenetically, first with the NJ method and finally with the maximum likelihood (ML) method. The tree thus obtained (Figure 1) shows clearly that the sequences cluster in four major clades, each one supported by high significance. The sequence used as out-group to root the tree was the amphioxus QRFP receptor (Xu et al., submitted) which in our previous calculations, using more distantly related sequences as out-group, would always branch off basally to all of the vertebrate QRFPR sequences.

No single species contains all four genes. Nevertheless, by combining information from the 20 species included in the tree, the taxonomic distribution implies that four genes existed in early vertebrate evolution. The largest clade is the one containing the human QRFP receptor, henceforce called QRFPR1 to distinguish it from the other subtypes. Orthologs of this gene exist in the mammals included in the analysis, as well as in chicken and in representatives from three other major amniotes lineages, i.e., an alligator, a lizard, and a turtle (Figure 1). This subtype is also found in the frog and the coelacanth.

The smallest clade is the one given the subtype name QRFPR2 which is present in gar, coelacanth, frog, and turtle. The two former species also possess QRFPR4 which is also found in three of the teleost fish species. Also the single receptor sequence of the sea lamprey, *Petromyzon marinus*, belongs to this clade with high statistical support. The final clade, QRFPR3, is present in all ray-finned fish species investigated, both the spotted gar and all of the teleosts. Finally, it should be added that both the alligator and the lizard have a second receptor gene, which however could not be assigned to a specific subtype due to incomplete information in the sequence databases. Two of the zebrafish sequences cluster close to each other in the QRFPR3 clade, supporting duplication in conjunction with the teleost 3R event.

### Synteny and paralogon analyses of QRFPR sequences

**Figure 2:**
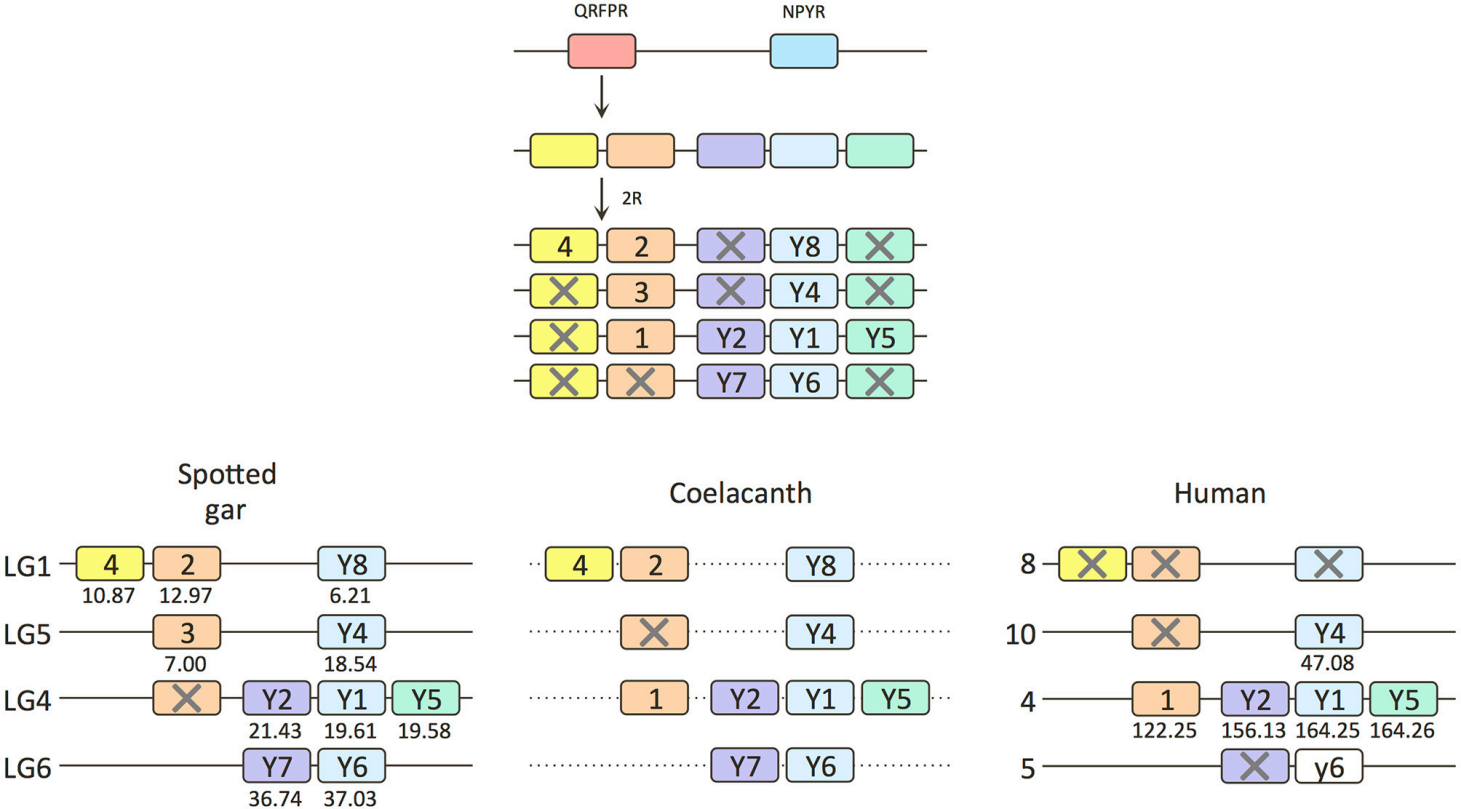
**Gene duplication events for the QRFP receptor family in early vertebrate evolution including local duplications and the two basal vertebrate tetraploidizations**. NPYR stands for neuropeptide Y receptors. Crosses mark gene losses. The human Y6 gene is a pseudogene. The dashed lines for the coelacanth indicate that it is not known if the genes are syntenic. Numbers under gene boxes show map positions along the chromosomes in megabase pairs.

The presence of four QRFPR clades naturally suggests origin by duplications resulting from the two basal tetraploidizations, 1R and 2R, thus forming a paralogon (a set of chromosome regions containing members of the same gene families as a result of duplication of a large block or an entire chromosome). However, due to the many losses of QRFPR genes in the different species or lineages, sequence-based analyses may be skewed due to uneven selection pressures. Therefore, some additional type of information should be considered. The human QRFPR1 gene is located on chromosome 4, the same chromosome that contains no less than three NPY-family receptors, namely subtypes 1, 2, and 5 (Figure 2). This synteny suggests that QRFPR1 arose as a duplicate of the ancestral NPYR (or the other way around). As the NPYR triplet was clearly quadrupled in the 1R-2R events (Larhammar and Salaneck, 2004; Larhammar and Bergqvist, 2013), this suggests that QRFPR1 could have been duplicated simultaneously. Therefore, we have analyzed the chromosomal locations of the other QRFPR genes, especially in the species which is known to have a genome with very few chromosomal rearrangements, the spotted gar (Amores et al., 2011).

Two of the receptors in the spotted gar, QRFPR2 and 4, are located on linkage group 1 (LG1) which also contains NPY8R (Figure 2). All three of these genes are missing in the human genome, but NPY8R is known to be in a chromosome region in teleost fishes (Larsson et al., 2008) that displays conserved synteny with human chromosome 8 (abbreviated Hsa8 for *Homo sapiens* chromosome 8). The coelacanth has all three of these genes, NPY8R, and QRFPR2 and 4, but they are located on separate scaffolds.

The gar QRFPR3 gene is located on LG5 together with NPY4R which in human is on Hsa10, also a member of this paralogon (Figure 2). The QRFPR1 subtype found in mammals is unfortunately missing in the gar, but as this gene is located on Hsa4 together with the NPY receptor cluster, which is present in gar on LG4, the missing QRFPR1 gene probably once resided there too.

The chromosome harboring NPY receptors Y6 and Y7 in gar (Figure 2) as well as chicken (Larsson et al., 2009) corresponds to Hsa5 where the NPY6R pseudogene is located. NPY7R was lost in the mammalian ancestor. No QRFPR subtype has been identified in any species that might correspond to a gene located on this chromosome.

The two zebrafish QRFPR genes belonging to the QRFPR3 clade are located on zebrafish chromosomes 12 and 13 (Supplementary Table 1). These are known to be 3R duplicates of the chromosome corresponding to Hsa10 containing NPY4R (Larsson et al., 2008) and to gar LG5, hence the zebrafish genes have been named QRFPR3a and b.

## Discussion

The high statistical support for the four clades in Figure 1, and the species distribution of the receptors, argues strongly for an ancestral quartet of QRFP receptors although no single extant species or lineages is in possession of all four subtypes. The two crucial vertebrate lineages are represented by the spotted gar, an early branching ray-finned fish, and the African coelacanth, or latimeria, an early branch in the clade of lobe-finned fishes (Sarcopterygii) including also lungfishes and tetrapods. Both the gar and the coelacanth have been reported to evolve more slow than several other lineages, the gar by having fewer chromosomal rearrangements and the coelacanth by having fewer amino acid changes in many proteins. Therefore, it is of great importance that we found three QRFPR genes in both of these species, two of which are orthologs, namely QRFPR2 and QRFPR4. The remaining subtypes are found in each of these two species, QRFPR1 in coelacanth and QRFPR3 in gar. Taken together, this information points to four distinct receptor subtypes before the divergence of ray-finned and lobe-finned fishes.

As the QRFPR1 gene in human (and other mammals) and the QRFPR2 and QRFPR3 genes in gar are located in chromosome regions that belong to the well-characterized paralogon encoding the seven NPY receptor subtypes, which is known to have undergone chromosome duplications in 2R (Larhammar and Salaneck, 2004; Larsson et al., 2008, 2009; Larhammar and Bergqvist, 2013), this argues strongly for origin of these three QRFPR subtypes as a result of chromosome duplications in 1R and 2R, and by extension the origin of zebrafish QRFPR3a and 3b in the teleost 3R tetraploidization.

The origin of the QRFPR4 gene is more difficult to deduce. It has broad taxonomic distribution, being present in both coelacanth, gar, and teleost fishes, as well as the sea lamprey. In the phylogenetic tree it branches off with high statistical support prior to the radiation of the other three subtypes. As the QRFPR4 gene is located in the same linkage group as QRFPR2 in the gar, this indicates origin of the two by duplication from a common ancestor. If so, the phylogenetic tree implies that this duplication took place prior to the chromosome duplications in 1R and 2R. This would mean that any 1R/2R duplicates of QRFPR4 have been lost from the chromosomes corresponding to gar LG4, 5, and 6.

Another possibility is that QRFPR4 is actually the fourth and missing member of the QRFP1,2,3 quartet resulting from 2R, but has been translocated from the chromosome corresponding to LG6 (which presently lacks a QRFP receptor) onto LG1 near the QRFPR2 gene. Such translocations by unequal crossing over were probably more likely to happen soon after 2R while the quadrupled chromosomes were still highly identical to each other. This scenario would require that QRFPR4 has had a higher evolutionary rate, to explain its basal position in the three. Unfortunately the coelacanth scaffolds are not large enough to say whether QRFPR2 and 4 belong on the same chromosome. More detailed analyses of the synteny groups of all of the QRFP receptor genes may resolve these alternative scenarios. Unfortunately our searches in the genome of a cartilaginous fish, the elephant shark *Callorhinchus milii*, has so far yielded no certain QRFP receptor sequences, nor a QRFP peptide precursor gene. More extensive studies are required before conclusions can be drawn about possible loss of the entire QRFP system in chimeras or the entire class of Chondrichthyes.

Analyses of teleost fish chromosomes is more complicated due to the high frequency of rearrangements, probably as a result of the teleost 3R tetraploidization. However, it is obvious that very few 3R duplicates of the QRFPR genes have survived. In fact, the zebrafish QRFPR3a and 3b are the only ones in the entire QRFPR tree. Also some other GPCR families show this remarkable degree of gene loss after 3R, for instance the NPY receptor family where only a singly 3R duplicate has survived, namely for NPY8R (Larsson et al., 2008). For the distantly related families of kisspeptin receptors and visual opsins, not a single one of the four and five ancestral genes, respectively, have survived 3R (Pasquier et al., 2012; Lagman et al., 2013). In contrast, some GPCR families gained multiple members in 3R: in the somatostatin receptor 2-3-5 family, all three members were duplicated (Ocampo Daza et al., 2012), for the endothelin receptors two out of three members have 3R duplicates (Braasch and Schartl, 2014), and for the opioid receptor family two of the four ancestral receptors have 3R duplicates (Dreborg et al., 2008).

Although the coelacanth has been found to have a slow pace of evolution (Amemiya et al., 2013), some genes seem to have evolved more rapidly in this lineage. Interestingly, this seems to concern QRFPR1 as indicated by its long branch, as seems to be the case also for gar QRFPR2 (Figure 1). More detailed scrutiny of the receptor sequences may give clues as to what aspects of receptor function might be affected by the increased evolutionary rate.

Despite the presence of four QRFPR subtypes in the ancestral vertebrate after 2R, there are no reports about additional copies of the QRFP peptide gene. This implies that this peptide alone served as ligand on the four ancestral receptors. On the other hand, amphioxus seems to have three QRFP-related peptides (Mirabeau and Joly, 2013) and a single receptor (Xu et al., submitted). However, it remains to be investigated if all three peptides act on this receptor.

In conclusion, the QRFP receptor family shows considerable complexity with four members in the ancestral vertebrate and three members still existing in spotted gar and coelacanth. This invites to further studies of this peptide-receptor system in these species, as well as many other species with more than just the single receptor found in mammals.

### Conflict of interest statement

The authors declare that the research was conducted in the absence of any commercial or financial relationships that could be construed as a potential conflict of interest.

## Supplementary material

Supplementary Figure 1 **Alignment of the QRFP receptor sequences used for calculating the phylogenetic tree in Figure 1**.

Supplementary Table 1 **Ensemble gene ID or NCBI accession numbers for vertebrate QRFP receptors**. As indicated in the right hand column, the annotation of many of the entries had to be curated by manual editing.
