# Supplementary material for: Unexpected multiplicity of QRFP receptors in early vertebrate evolution
Source: Front Neurosci. 2014 Oct 24;8:337. doi: 10.3389/fnins.2014.00337 (PMC4208404; doi:10.3389/fnins.2014.00337)
Supplement: Supplementary Figure 1 — Alignment of the QRFP receptor sequences used for calculating the phylogenetic tree in Figure 1. [file Image1.PDF]

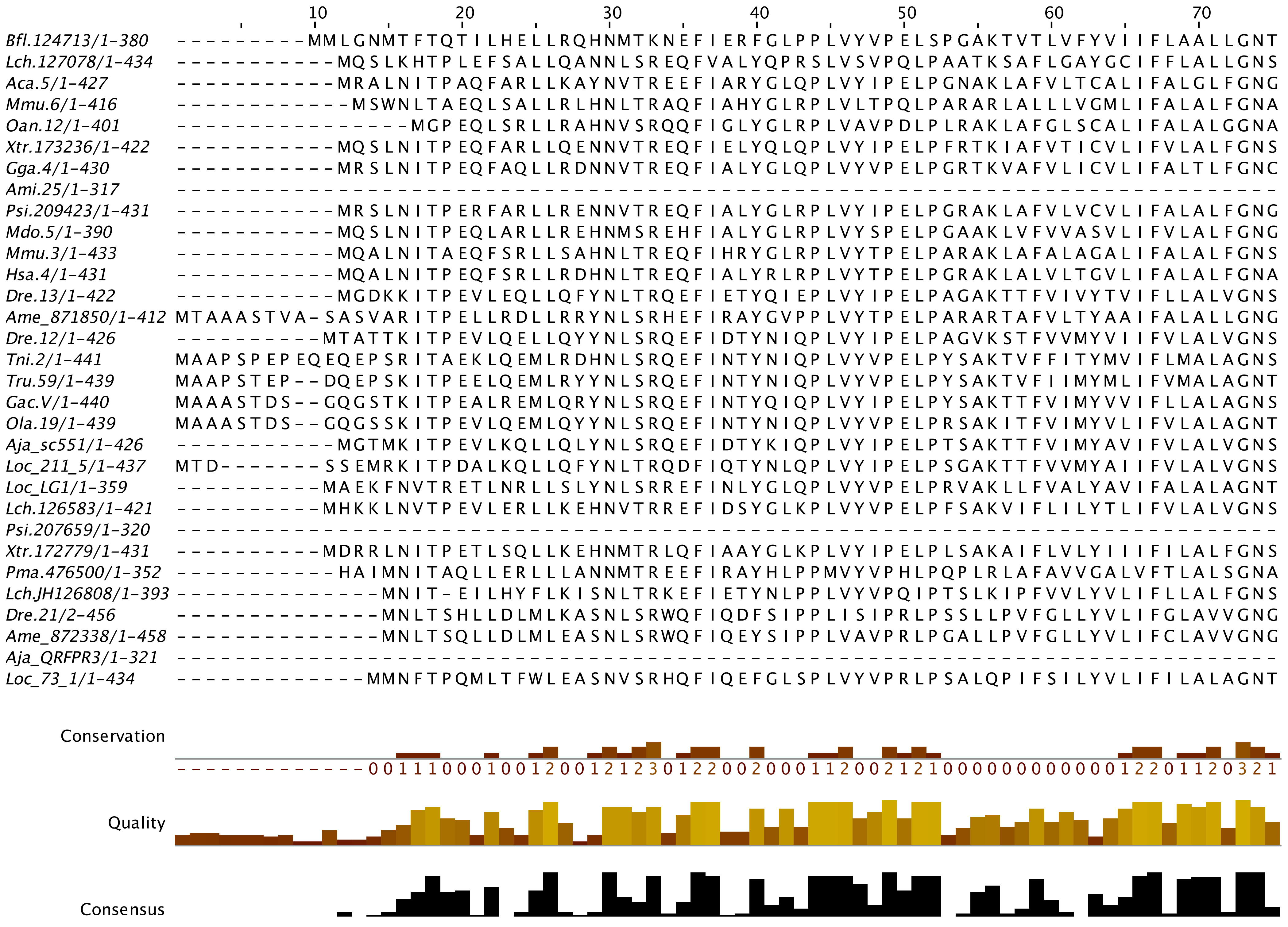
10 20 30 40 50 60 70
Bfl.124713/1-380 ---------MMLGNMTFTQTILHELLRQHNMTKNEFIERFGLPPLVYVPELSPGAKTVTLVFYVIIFLAALLGNT
Lch.127078/1-434 -----------MQSLKHTPLEFSALLQANNLSREQFVALYQPRSLVSVPQLPAATKSAFLGAYGCIFFLALLGNS
Aca.5/1-427 -----------MRALNITPAQFARLLKAYNVTREEFIARYGLQPLVYIPELPGNAKLAFVLTCALIFALGLFGNG
Mmu.6/1-416 ------------MSWNLTAEQLSALLRLHNLTRAQFIAHYGLRPLVLTPQLPARARLALLLVGMLIFALALFGNA
Oan.12/1-401 ----------------MGPEQLSRLLRAHNVSRQQFIGLYGLRPLVAVPDLPLRAKLAFGLSCALIFALALGGNA
Xtr.173236/1-422 -----------MQSLNITPEQFARLLQENNVTREQFIELYQLQPLVYIPELPFRTKIAFVTICVLIFVLALFGNS
Gga.4/1-430 -----------MRSLNITPEQFAQLLRDNNVTREQFIALYGLQPLVYVPELPGRTKVAFVLICVLIFALTLFGNC
Ami.25/1-317 ---
Psi.209423/1-431 -----------MRSLNITPERFARLLRENNVTREQFIALYGLRPLVYIPELPGRAKLAFVLVCVLIFALALFGNG
Mdo.5/1-390 -----------MQSLNITPEQLARLLREHNMSREHFIALYGLRPLVYSPELPGAAKLVFVVASVLIFVLALFGNG
Mmu.3/1-433 -----------MQALNITAEQFSRLLSAHNLTREQFIHRYGLRPLVYTPELPARAKLAFALAGALIFALALFGNS
Hsa.4/1-431 -----------MQALNITPEQFSRLLRDHNLTREQFIALYRLRPLVYTPELPGRAKLALVLTGVLIFALALFGNA
Dre.13/1-422 -----------MGDKKITPEVLEQLLQFYNLTRQEFIETYQIEPLVYIPELPAGAKTTFVIVYTVIFLLALVGNS
Ame_871850/1-412 MTAAASTVA-SASVARITPELLRDLLRRYNLSRHEFIRAYGVPPLVYTPELPARARTAFVLTYAAIFALALLGNG
Dre.12/1-426 ----------MTATTKITPEVLQELLQYYNLSRQEFIDTYNIQPLVYIPELPAGVKSTFVVMYVIIFVLALVGNS
Tni.2/1-441 MAAPSPEPEQEQEPSRITAEKLQEMLRDHNLSRQEFINTYNIQPLVYVPELPYSAKTVFFITYMVIFLMALAGNS
Tru.59/1-439 MAAPSTEP--DQEPSKITPEELQEMLRYYNLSRQEFINTYNIQPLVYVPELPYSAKTVFIIMYMLIFVMALAGNT
Gac.V/1-440 MAAASTDS--GQGSTKITPEALREMLQRYNLSRQEFINTYQIQPLVYIPELPYSAKTTFVIMYVVIFLLALAGNS
Ola.19/1-439 MAAASTDS--GQGSSKITPEVLQEMLQYYNLSRQEFINTYNIQPLVYVPELPRSAKITFVIMYVLIFVLALAGNT
Aja_sc551/1-426 -----------MGTMKITPEVLKQLLQLYNLSRQEFIDTYKIQPLVYIPELPTSAKTTFVIMYAVIFVLALVGNS
Loc_211_5/1-437 MTD-------SSEMRKITPDALKQLLQFYNLTRQDFIQTYNLQPLVYIPELPSGAKTTFVVMYAIIFVLALVGNS
Loc_LG1/1-359 ----------MAEKFNVTRETLNRLLSLYNLSRREFINLYGLQPLVYVPELPRVAKLLFVALYAVIFALALAGNT
Lch.126583/1-421 ----------MHKKLNVTPEVLERLLKEHNVTRREFIDSYGLKPLVYVPELPFSAKVIFLILYTLIFVLALVGNS
Psi.207659/1-320 ---
Xtr.172779/1-431 ----------MDRRLNITPETLSQLLKEHNMTRLQFIAAYGLKPLVYIPELPLSAKAIFLVLYIIIFILALFGNS
Pma.476500/1-352 -----------HAIMNITAQLLERLLLANNMTREEFIRAYHLPPMVYVPHLPQPLRLAFAVVGALVFTLALSGNA
Lch.JH126808/1-393 --------------MNIT-EILHYFLKISNLTRKEFIETYNLPPLVYVPQIPTSLKIPFVVLYVLIFLLALFGNS
Dre.21/2-456 --------------MNLTSHLLDLMLKASNLSRWQFIQDFSIPPLISIPRLPSSLLPVFGLLYVLIFGLAVVGNG
Ame_872338/1-458 --------------MNLTSQLLDLMLEASNLSRWQFIQEYSIPPLVAVPRLPGALLPVFGLLYVLIFCLAVVGNG
Aja_QRFPR3/1-321 ---
Loc_73_1/1-434 -------------MMNFTPQMLTFWLEASNVSRHQFIQEFGLSPLVYVPRLPSALQPIFSILYVLIFILALAGNT
Conservation
-------------00111000100120012123012200200011200212100000000000012201120321
Quality
Consensus

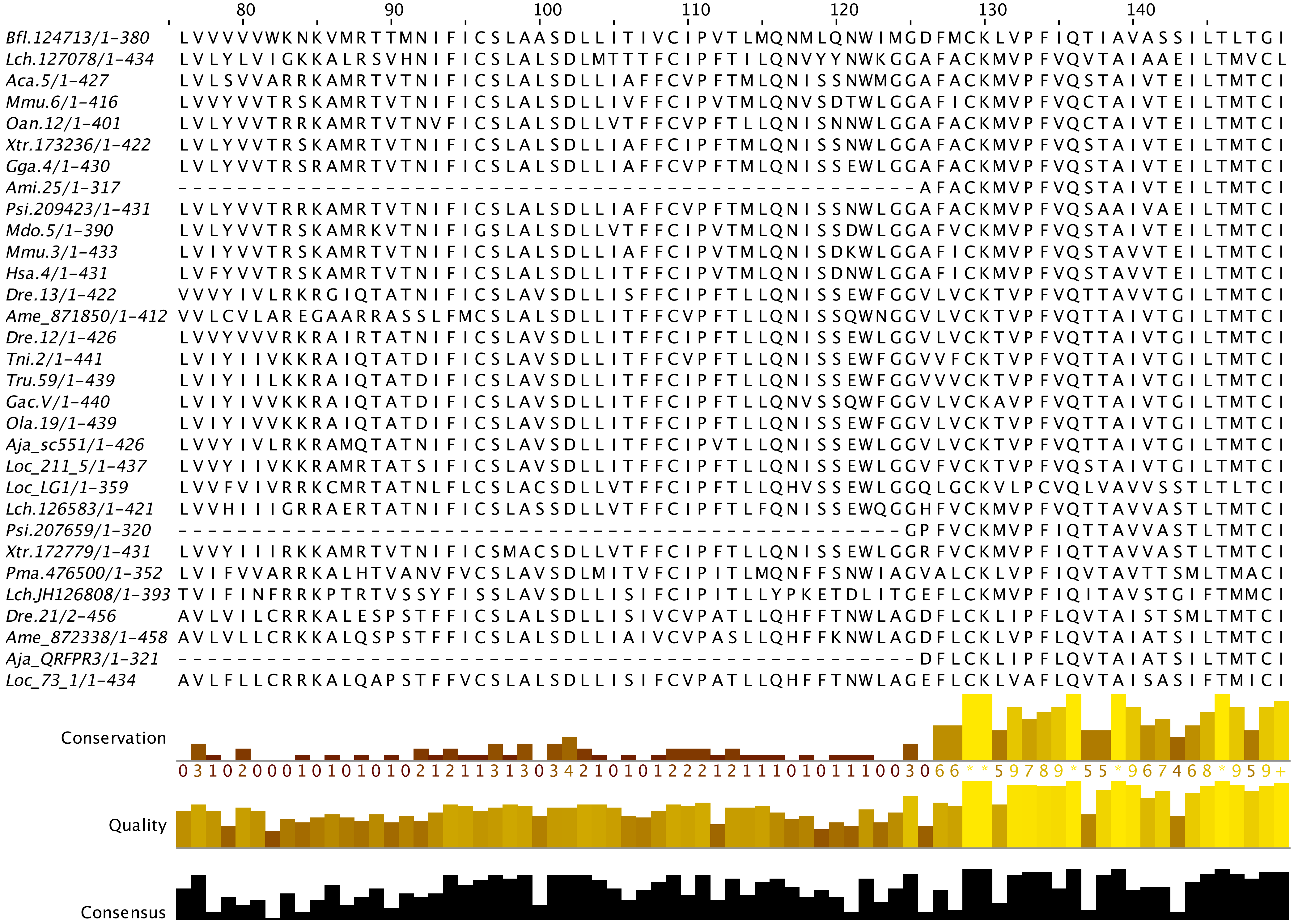

80 90 100 110 120 130 140
Bfl.124713/1-380 LVVVVVWKNKVMRTTMNIFICSLAASDLLITIVCIPVTLMQNMLQNWIMGDFMCKLVPFIQTIAVASSILTLTGI
Lch.127078/1-434 LVLYLVIGKKALRSVHNIFICSLALSDLMTTTFCIPFTILQNVYYNWKGGAFACKMVPFVQVTAIAAEILTMVCL
Aca.5/1-427 LVLSVVARRKAMRTVTNIFICSLALSDLLIAFFCVPFTMLQNISSNWMGGAFACKMVPFVQSTAIVTEILTMTCI
Mmu.6/1-416 LVVYVVTRSKAMRTVTNIFICSLALSDLLIVFFCIPVTMLQNVSDTWLGGAFICKMVPFVQCTAIVTEILTMTCI
Oan.12/1-401 LVLYVVTRRKAMRTVTNVFICSLALSDLLVTFFCVPFTLLQNISNNWLGGAFACKMVPFVQCTAIVTEILTMTCI
Xtr.173236/1-422 LVLYVVTRSKAMRTVTNIFICSLALSDLLIAFFCIPFTMLQNISSNWLGGAFACKMVPFVQSTAIVTEILTMTCI
Gga.4/1-430 LVLYVVTRSRAMRTVTNIFICSLALSDLLIAFFCVPFTMLQNISSEWLGGAFACKMVPFVQSTAIVTEILTMTCI
Ami.25/1-317 --AFACKMVPFVQSTAIVTEILTMTCI
Psi.209423/1-431 LVLYVVTRRKAMRTVTNIFICSLALSDLLIAFFCVPFTMLQNISSNWLGGAFACKMVPFVQSAAIVAEILTMTCI
Mdo.5/1-390 LVLYVVTRSKAMRKVTNIFIGSLALSDLLVTFFCIPVTMLQNISSDWLGGAFVCKMVPFVQSTAIVTEILTMTCI
Mmu.3/1-433 LVIYVVTRSKAMRTVTNIFICSLALSDLLIAFFCIPVTMLQNISDKWLGGAFICKMVPFVQSTAVVTEILTMTCI
Hsa.4/1-431 LVFYVVTRSKAMRTVTNIFICSLALSDLLITFFCIPVTMLQNISDNWLGGAFICKMVPFVQSTAVVTEILTMTCI
Dre.13/1-422 VVVYIVLRKRGIQTATNIFICSLAVSDLLISFFCIPFTLLQNISSEWFGGVLVCKTVPFVQTTAVVTGILTMTCI
Ame_871850/1-412 VVLCVLAREGAARRASSLFMCSLALSDLLITFFCVPFTLLQNISSQWNGGVLVCKTVPFVQTTAIVTGILTMTCI
Dre.12/1-426 LVVYVVVRKRAIRTATNIFICSLAVSDLLITFFCIPFTLLQNISSEWLGGVLVCKTVPFVQTTAIVTGILTMTCI
Tni.2/1-441 LVIYIIVKKRAIQTATDIFICSLAVSDLLITFFCVPFTLLQNISSEWFGGVVFCKTVPFVQTTAIVTGILTMTCI
Tru.59/1-439 LVIYIILKKRAIQTATDIFICSLAVSDLLITFFCIPFTLLQNISSEWFGGVVVCKTVPFVQTTAIVTGILTMTCI
Gac.V/1-440 LVIYIVVKKRAIQTATDIFICSLAVSDLLITFFCIPFTLLQNVSSQWFGGVLVCKAVPFVQTTAIVTGILTMTCI
Ola.19/1-439 LVIYIVVKKRAIQTATDIFICSLAVSDLLITFFCIPFTLLQNISSEWFGGVLVCKTVPFVQTTAIVTGILTMTCI
Aja_sc551/1-426 LVVYIVLRKRAMQTATNIFICSLAVSDLLITFFCIPVTLLQNISSEWLGGVLVCKTVPFVQTTAIVTGILTMTCI
Loc_211_5/1-437 LVVYIIVKKRAMRTATSIFICSLAVSDLLITFFCIPFTLLQNISSEWLGGVFVCKTVPFVQSTAIVTGILTMTCI
Loc_LG1/1-359 LVVFVIVRRKCMRTATNLFLCSLACSDLLVTFFCIPFTLLQHVSSEWLGGQLGCKVLPCVQLVAVVSSTLTLTCI
Lch.126583/1-421 LVVHIIIGRRAERTATNIFICSLASSDLLVTFFCIPFTLFQNISSEWQGGHFVCKMVPFVQTTAVVASTLTMTCI
Psi.207659/1-320 ---GPFVCKMVPFIQTTAVVASTLTMTCI
Xtr.172779/1-431 LVVYIIIRKKAMRTVTNIFICSMACSDLLVTFFCIPFTLLQNISSEWLGGRFVCKMVPFIQTTAVVASTLTMTCI
Pma.476500/1-352 LVIFVVARRKALHTVANVFVCSLAVSDLMITVFCIPITLMQNFFSNWIAGVALCKLVPFIQVTAVTTSMLTMACI
Lch.JH126808/1-393 TVIFINFRRKPTRTVSSYFISSLAVSDLLISIFCIPITLLYPKETDLITGEFLCKMVPFIQITAVSTGIFTMMCI
Dre.21/2-456 AVLVILCRRKALESPSTFFICSLALSDLLISIVCVPATLLQHFFTNWLAGDFLCKLIPFLQVTAISTSMLTMTCI
Ame_872338/1-458 AVLVLLCRKKALQSPSTFFICSLALSDLLIAIVCVPASLLQHFFKNWLAGDFLCKLVPFLQVTAIATSILTMTCI
Aja_QRFPR3/1-321 --DFLCKLIPFLQVTAIATSILTMTCI
Loc_73_1/1-434 AVLFLLCRRKALQAPSTFFVCSLALSDLLISIFCVPATLLQHFFTNWLAGEFLCKLVAFLQVTAISASIFTMICI
Conservation
0310200010101010212113130342101012221211101011100306 6**59789*55*967468*959+
Quality
Consensus

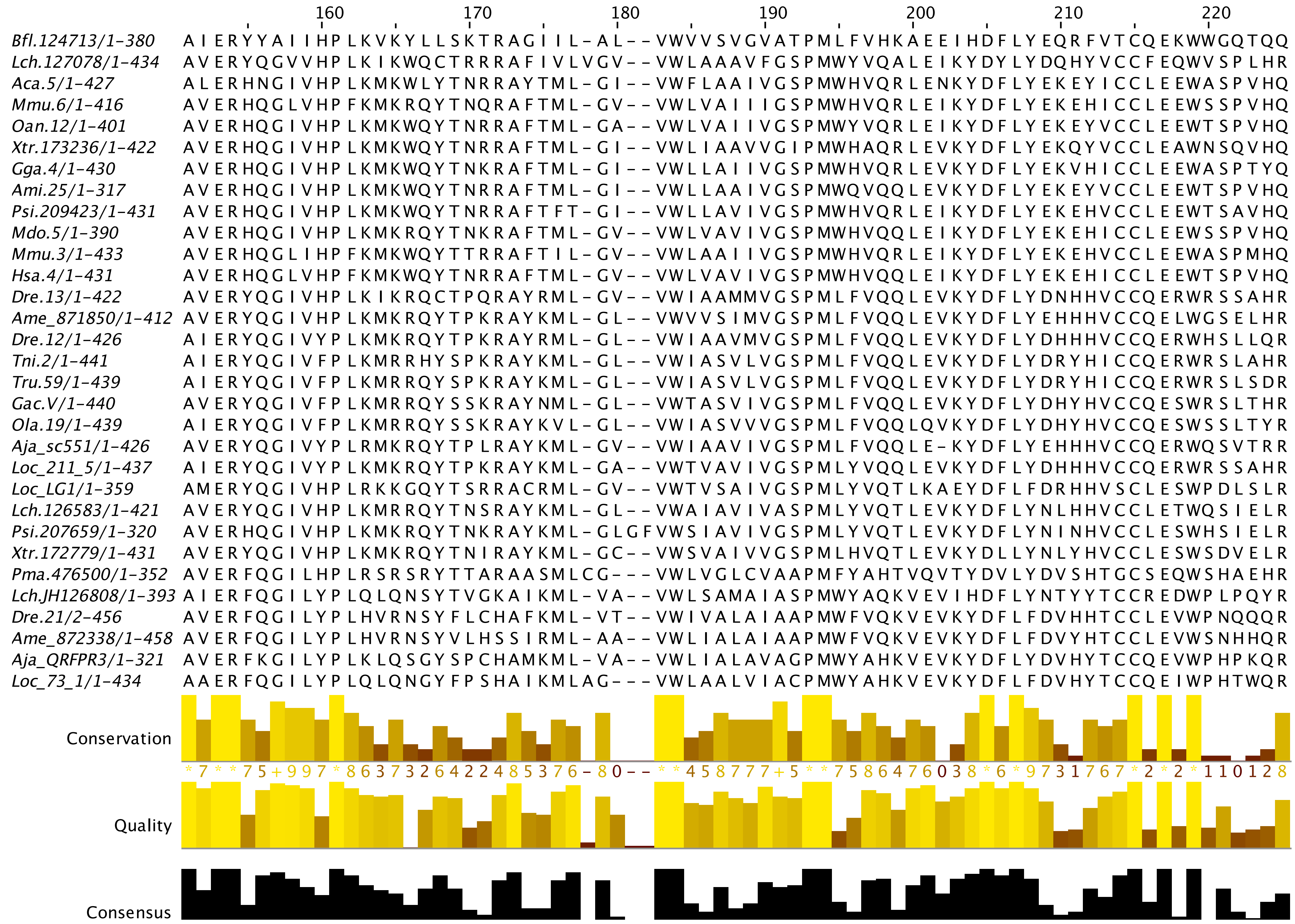
160 170 180 190 200 210 220
Bfl.124713/1-380 AIERYYAIIHPLKVKYLLSKTRAGIIL-AL--VWVVSVGVATPMLFVHKAEEIHDFLYEQRFVTCQEKWWGQTQQ
Lch.127078/1-434 AVERYQGVVHPLKIKWQCTRRRAFIVLVGV--VWLAAAVFGSPMWYVQALEIKYDYLYDQHYVCCFEQWVSPLHR
Aca.5/1-427 ALERHNGIVHPLKMKWLYTNRRAYTML-GI--VWFLAAIVGSPMWHVQRLENKYDFLYEKEYICCLEEWASPVHQ
Mmu.6/1-416 AVERHQGLVHPFKMKRQYTNQRAFTML-GV--VWLVAIIIGSPMWHVQRLEIKYDFLYEKEHICCLEEWSSPVHQ
Oan.12/1-401 AVERHQGIVHPLKMKWQYTNRRAFTML-GA--VWLVAIIVGSPMWYVQRLEIKYDFLYEKEYVCCLEEWTSPVHQ
Xtr.173236/1-422 AVERHQGIVHPLKMKWQYTNRRAFTML-GI--VWLIAAVVGIPMWHAQRLEVKYDFLYEKQYVCCLEAWNSQVHQ
Gga.4/1-430 AVERHQGIVHPLKMKWQYTNKRAFTML-GI--VWLLAIIVGSPMWHVQRLEVKYDFLYEKVHICCLEEWASPTYQ
Ami.25/1-317 AVERHQGIVHPLKMKWQYTNRRAFTML-GI--VWLLAAIVGSPMWQVQQLEVKYDFLYEKEYVCCLEEWTSPVHQ
Psi.209423/1-431 AVERHQGIVHPLKMKWQYTNRRAFTFT-GI--VWLLAVIVGSPMWHVQRLEIKYDFLYEKEHVCCLEEWTSAVHQ
Mdo.5/1-390 AVERHQGIVHPLKMKRQYTNKRAFTML-GV--VWLVAVIVGSPMWHVQQLEIKYDFLYEKEHICCLEEWSSPVHQ
Mmu.3/1-433 AVERHQGLIHPFKMKWQYTTRRAFTIL-GV--VWLAAIIVGSPMWHVQRLEIKYDFLYEKEHVCCLEEWASPMHQ
Hsa.4/1-431 AVERHQGLVHPFKMKWQYTNRRAFTML-GV--VWLVAVIVGSPMWHVQQLEIKYDFLYEKEHICCLEEWTSPVHQ
Dre.13/1-422 AVERYQGIVHPLKIKRQCTPQRAYRML-GV--VWIAAMMVGSPMLFVQQLEVKYDFLYDNHHVCCQERWRSSAHR
Ame_871850/1-412 AVERYQGIVHPLKMKRQYTPKRAYKML-GL--VWVVSIMVGSPMLFVQQLEVKYDFLYEHHHVCCQELWGSELHR
Dre.12/1-426 AIERYQGIVYPLKMKRQYTPKRAYRML-GL--VWIAAVMVGSPMLFVQQLEVKYDFLYDHHHVCCQERWHSLLQR
Tni.2/1-441 AIERYQGIVFPLKMRRHYSPKRAYKML-GL--VWIASVLVGSPMLFVQQLEVKYDFLYDRYHICCQERWRSLAHR
Tru.59/1-439 AIERYQGIVFPLKMRRQYSPKRAYKML-GL--VWIASVLVGSPMLFVQQLEVKYDFLYDRYHICCQERWRSLSDR
Gac.V/1-440 AVERYQGIVFPLKMRRQYSSKRAYNML-GL--VWTASVIVGSPMLFVQQLEVKYDFLYDHYHVCCQESWRSLTHR
Ola.19/1-439 AIERYQGIVFPLKMRRQYSSKRAYKVL-GL--VWIASVVVGSPMLFVQQLQVKYDFLYDHYHVCCQESWSSLTYR
Aja_sc551/1-426 AVERYQGIVYPLRMKRQYTPLRAYKML-GV--VWIAAVIVGSPMLFVQQLE-KYDFLYEHHHVCCQERWQSVTRR
Loc_211_5/1-437 AIERYQGIVYPLKMKRQYTPKRAYKML-GA--VWTVAVIVGSPMLYVQQLEVKYDFLYDHHHVCCQERWRSSAHR
Loc_LG1/1-359 AMERYQGIVHPLRKKGQYTSRRACRML-GV--VWTVSAIVGSPMLYVQTLKAEYDFLFDRHHVSCLESWPDLSLR
Lch.126583/1-421 AVERYQGIVHPLKMRRQYTNSRAYKML-GL--VWAIAVIVASPMLYVQTLEVKYDFLYNLHHVCCLETWQSIELR
Psi.207659/1-320 AVERHQGIVHPLKMKRQYTNKRAYKML-GLGFVWSIAVIVGSPMLYVQTLEVKYDFLYNINHVCCLESWHSIELR
Xtr.172779/1-431 AVERYQGIVHPLKMKRQYTNIRAYKML-GC--VWSVAIVVGSPMLHVQTLEVKYDLLYNLYHVCCLESWSDVELR
Pma.476500/1-352 AVERFQGILHPLRSRSRYTTARAASMLCG---VWLVGLCVAAPMFYAHTVQVTYDVLYDVSHTGCSEQWSHAEHR
Lch.JH126808/1-393 AIERFQGILYPLQLQNSYTVGKAIKML-VA--VWLSAMAIASPMWYAQKVEVIHDFLYNTYYTCCREDWPLPQYR
Dre.21/2-456 AVERFQGILYPLHVRNSYFLCHAFKML-VT--VWIVALAIAAPMWFVQKVEVKYDFLFDVHHTCCLEVWPNQQQR
Ame_872338/1-458 AVERFQGILYPLHVRNSYVLHSSIRML-AA--VWLIALAIAAPMWFVQKVEVKYDFLFDVYHTCCLEVWSNHHQR
Aja_QRFPR3/1-321 AVERFKGILYPLKLQSGYSPCHAMKML-VA--VWLIALAVAGPMWYAHKVEVKYDFLYDVHYTCCQEVWPHPKQR
Loc_73_1/1-434 AAERFQGILYPLQLQNGYFPSHAIKMLAG---VWLAALVIACPMWYAHKVEVKYDFLFDVHYTCCQEIWPHTWQR
Conservation
*7**75+997*8637326422485376-80--**458777+5**7586476038*6*9731767*2*2*110128
Quality
Consensus

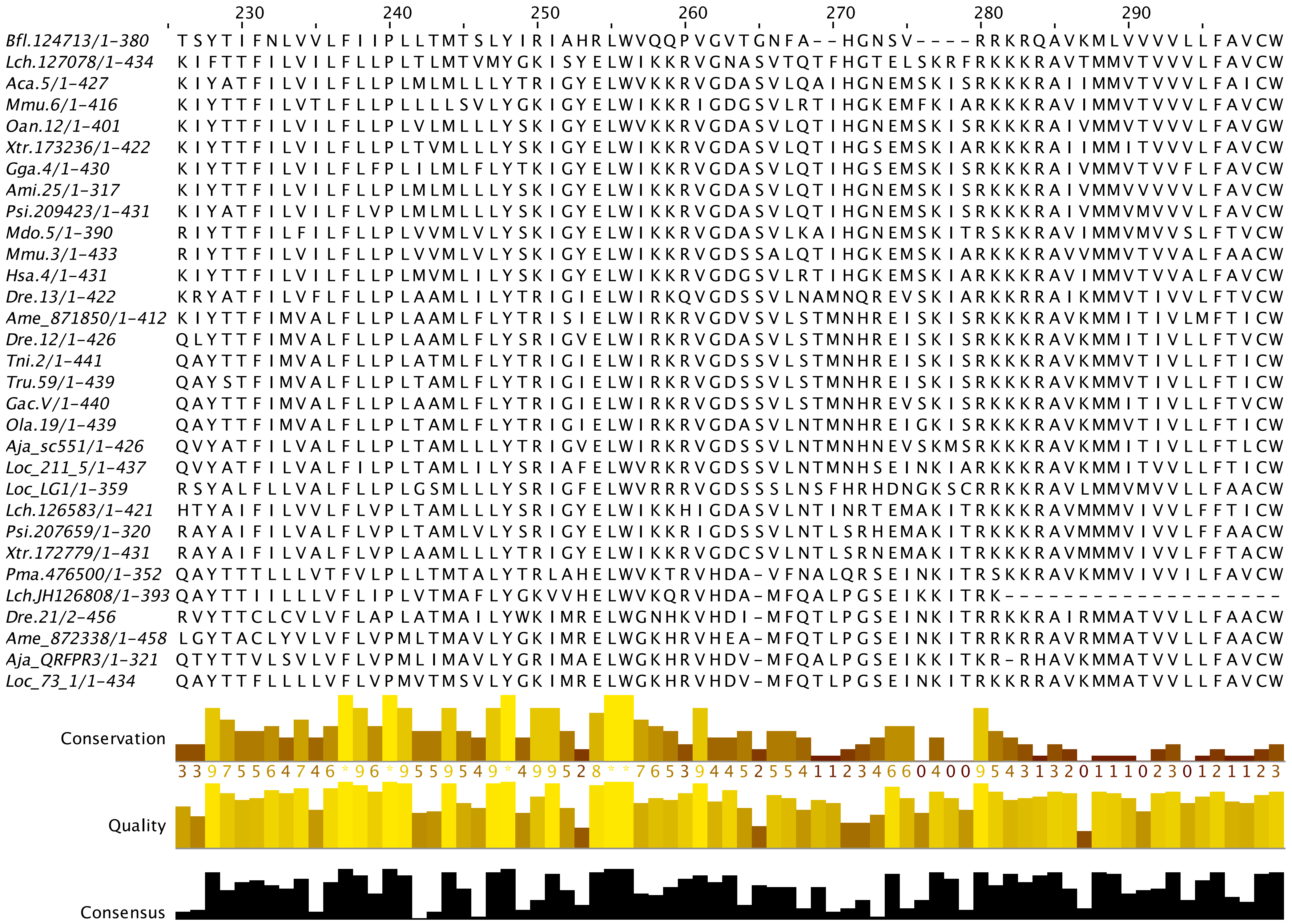

230 240 250 260 270 280 290
Bfl.124713/1-380
Lch.127078/1-434
Aca.5/1-427
Mmu.6/1-416
Oan.12/1-401
Xtr.173236/1-422
Gga.4/1-430
Ami.25/1-317
Psi.209423/1-431
Mdo.5/1-390
Mmu.3/1-433
Hsa.4/1-431
Dre.13/1-422
Ame_871850/1-412
Dre.12/1-426
Tni.2/1-441
Tru.59/1-439
Gac.V/1-440
Ola.19/1-439
Aja_sc551/1-426
Loc_211_5/1-437
Loc_LG1/1-359
Lch.126583/1-421
Psi.207659/1-320
Xtr.172779/1-431
Pma.476500/1-352
Lch.JH126808/1-393
Dre.21/2-456
Ame_872338/1-458
Aja_QRFPR3/1-321
Loc_73_1/1-434
Conservation
Quality
Consensus

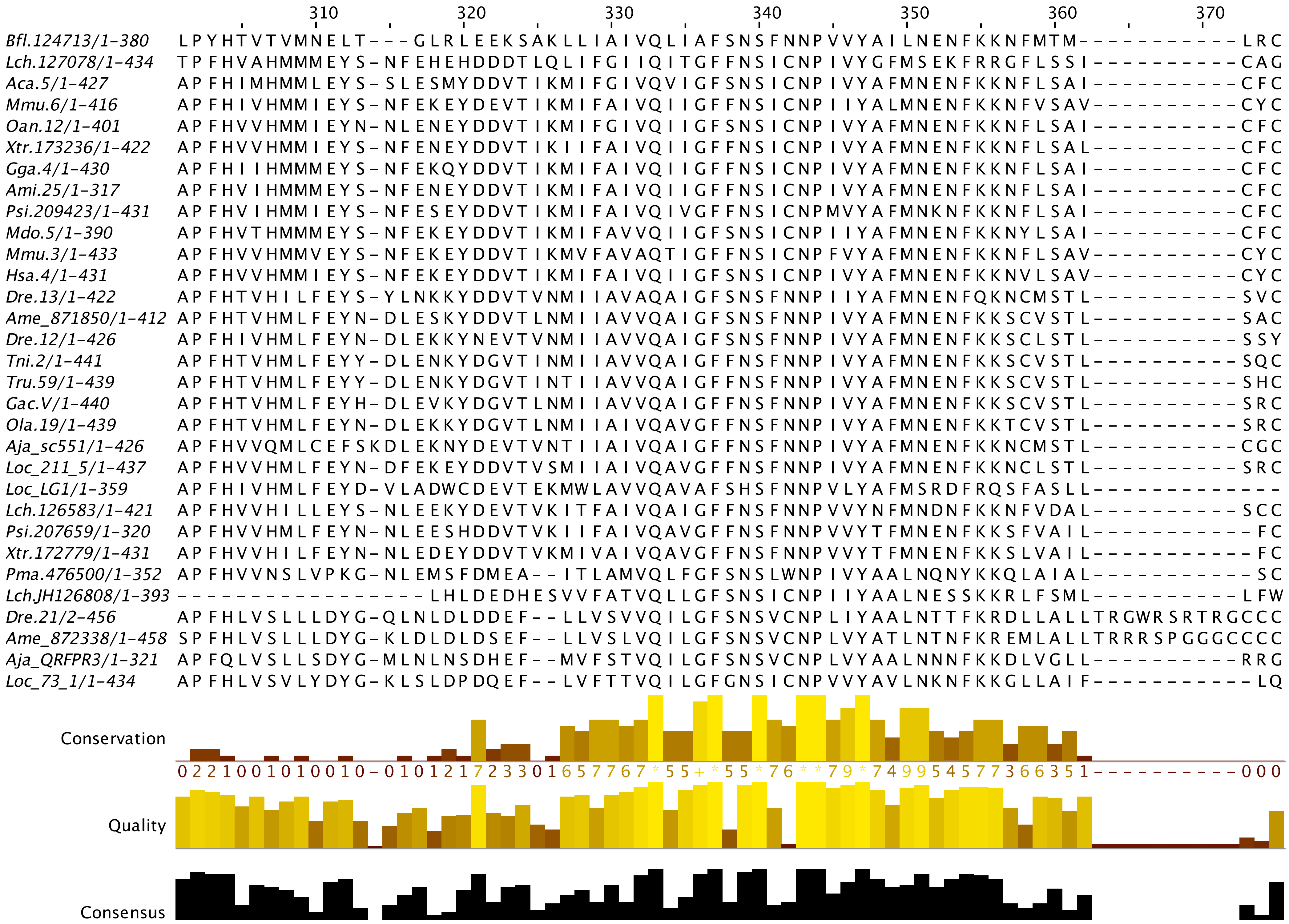
310 320 330 340 350 360 370
Bfl.124713/1-380 LPYHTVTVMNELT---GLRLEEKSAKLLIAIVQLIAFSNSFNNPVVYAILNENFKKNFMTM-----------LRC
Lch.127078/1-434 TPFHVAHMMMEYS-NFEHEHDDDTLQLIFGIIQITGFFNSICNPIVYGFMSEKFRRGFLSSI----------CAG
Aca.5/1-427 APFHIMHMMLEYS-SLESMYDDVTIKMIFGIVQVIGFSNSICNPIVYAFMNENFKKNFLSAI----------CFC
Mmu.6/1-416 APFHIVHMMIEYS-NFEKEYDEVTIKMIFAIVQIIGFFNSICNPIIYALMNENFKKNFVSAV----------CYC
Oan.12/1-401 APFHVVHMMIEYN-NLENEYDDVTIKMIFGIVQIIGFSNSICNPIVYAFMNENFKKNFLSAI----------CFC
Xtr.173236/1-422 APFHVVHMMIEYS-NFENEYDDVTIKIIFAIVQIIGFFNSICNPIVYAFMNENFKKNFLSAL----------CFC
Gga.4/1-430 APFHIIHMMMEYS-NFEKQYDDVTIKMIFAIVQIIGFFNSICNPIVYAFMNENFKKNFLSAI----------CFC
Ami.25/1-317 APFHVIHMMMEYS-NFENEYDDVTIKMIFAIVQIIGFFNSICNPIVYAFMNENFKKNFLSAI----------CFC
Psi.209423/1-431 APFHVIHMMIEYS-NFESEYDDVTIKMIFAIVQIVGFFNSICNPMVYAFMNKNFKKNFLSAI----------CFC
Mdo.5/1-390 APFHVTHMMMEYS-NFEKEYDDVTIKMIFAVVQIIGFSNSICNPIIYAFMNENFKKNYLSAI----------CFC
Mmu.3/1-433 APFHVVHMMVEYS-NFEKEYDDVTIKMVFAVAQTIGFFNSICNPFVYAFMNENFKKNFLSAV----------CYC
Hsa.4/1-431 APFHVVHMMIEYS-NFEKEYDDVTIKMIFAIVQIIGFSNSICNPIVYAFMNENFKKNVLSAV----------CYC
Dre.13/1-422 APFHTVHILFEYS-YLNKKYDDVTVNMIIAVAQAIGFSNSFNNPIIYAFMNENFQKNCMSTL----------SVC
Ame_871850/1-412 APFHTVHMLFEYN-DLESKYDDVTLNMIIAVVQAIGFSNSFNNPIVYAFMNENFKKSCVSTL----------SAC
Dre.12/1-426 APFHIVHMLFEYN-DLEKKYNEVTVNMIIAVVQAIGFSNSFNNPIVYAFMNENFKKSCLSTL----------SSY
Tni.2/1-441 APFHTVHMLFEYY-DLENKYDGVTINMIIAVVQAIGFFNSFNNPIVYAFMNENFKKSCVSTL----------SQC
Tru.59/1-439 APFHTVHMLFEYY-DLENKYDGVTINTIIAVVQAIGFFNSFNNPIVYAFMNENFKKSCVSTL----------SHC
Gac.V/1-440 APFHTVHMLFEYH-DLEVKYDGVTLNMIIAVVQAIGFFNSFNNPIVYAFMNENFKKSCVSTL----------SRC
Ola.19/1-439 APFHTVHMLFEYN-DLEKKYDGVTLNMIIAIVQAVGFFNSFNNPIVYAFMNENFKKTCVSTL----------SRC
Aja_sc551/1-426 APFHVVQMLCEFSKDLEKNYDEVTVNTIIAIVQAIGFFNSFNNPIVYAFMNENFKKNCMSTL----------CGC
Loc_211_5/1-437 APFHVVHMLFEYN-DFEKEYDDVTVSMIIAIVQAVGFFNSFNNPIVYAFMNENFKKNCLSTL----------SRC
Loc_LG1/1-359 APFHIVHMLFEYD-VLADWCDEVTEKMWLAVVQAVAFSHSFNNPVLYAFMSRDFRQSFASLL-------------
Lch.126583/1-421 APFHVVHILLEYS-NLEEKYDEVTVKITFAIVQAIGFFNSFNNPVVYNFMNDNFKKNFVDAL----------SCC
Psi.207659/1-320 APFHVVHMLFEYN-NLEESHDDVTVKIIFAIVQAVGFFNSFNNPVVYTFMNENFKKSFVAIL-----------FC
Xtr.172779/1-431 APFHVVHILFEYN-NLEDEYDDVTVKMIVAIVQAVGFFNSFNNPVVYTFMNENFKKSLVAIL-----------FC
Pma.476500/1-352 APFHVVNSLVPKG-NLEMSFDMEA--ITLAMVQLFGFSNSLWNPIVYAALNQNYKKQLAIAL-----------SC
Lch.JH126808/1-393 -----------------LHLDEDHESVVFATVQLLGFSNSICNPIIYAALNESSKKRLFSML----------LFW
Dre.21/2-456 APFHLVSLLLDYG-QLNLDLDDEF--LLVSVVQILGFSNSVCNPLIYAALNTTFKRDLLALLTRGWRSRTRGCCC
Ame_872338/1-458 SPFHLVSLLLDYG-KLDLDLDSEF--LLVSLVQILGFSNSVCNPLVYATLNTNFKREMLALLTRRRSPGGGCCCC
Aja_QRFPR3/1-321 APFQLVSLLSDYG-MLNLNSDHEF--MVFSTVQILGFSNSVCNPLVYAALNNNFKKDLVGLL----------RRG
Loc_73_1/1-434 APFHLVSVLYDYG-KLSLDPDQEF--LVFTTVQILGFGNSICNPVVYAVLNKNFKKGLLAIF-----------LQ
Conservation
0221001010010-0101217233016577767*55+*55*76**79*749954577366351---------000
Quality
Consensus

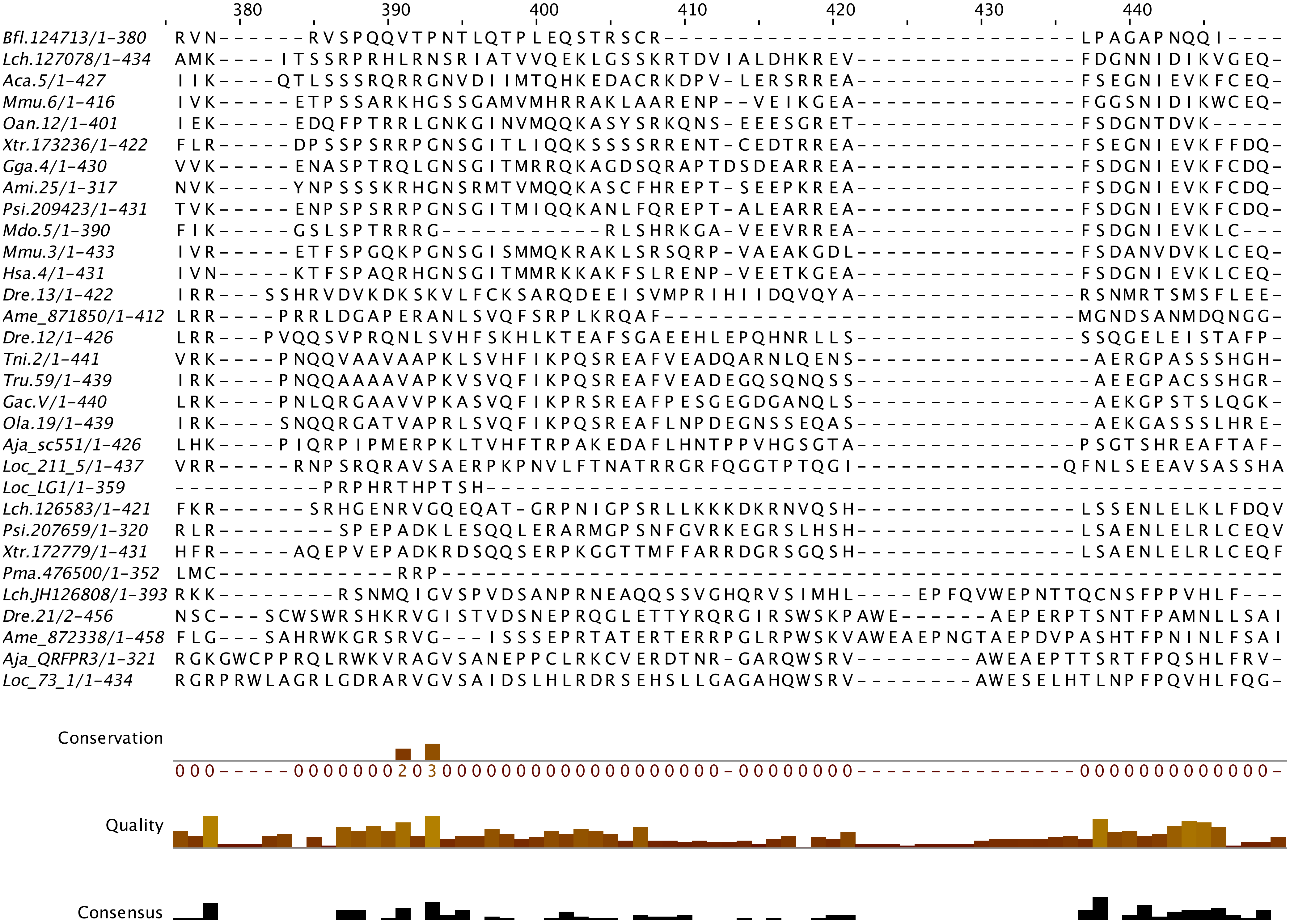
380 390 400 410 420 430 440
Bfl.124713/1-380 RVN------RVSPQQVTPNTLQTPLEQSTRSCR----------------------------LPAGAPNQQI----
Lch.127078/1-434 AMK----ITSSRPRHLRNSRIATVVQEKLGSSKRTDVIALDHKREV---------------FDGNNIDIKVGEQ-
Aca.5/1-427 IIK----QTLSSSRQRRGNVDIIMTQHKEDACRKDPV-LERSRREA---------------FSEGNIEVKFCEQ-
Mmu.6/1-416 IVK-----ETPSSARKHGSSGAMVMHRRAKLAARENP--VEIKGEA---------------FGGSNIDIKWCEQ-
Oan.12/1-401 IEK-----EDQFPTRRLGNKGINVMQQKASYSRKQNS-EEESGRET---------------FSDGNTDVK-----
Xtr.173236/1-422 FLR-----DPSSPSRRPGNSGITLIQQKSSSSRRENT-CEDTRREA---------------FSEGNIEVKFFDQ-
Gga.4/1-430 VVK-----ENASPTRQLGNSGITMRRQKAGDSQRAPTDSDEARREA---------------FSDGNIEVKFCDQ-
Ami.25/1-317 NVK-----YNPSSSKRHGNSRMTVMQQKASCFHREPT-SEEPKREA---------------FSDGNIEVKFCDQ-
Psi.209423/1-431 TVK-----ENPSPSRRPGNSGITMIQQKANLFQREPT-ALEARREA---------------FSDGNIEVKFCDQ-
Mdo.5/1-390 FIK-----GSLSPTRRRG-----------RLSHRKGA-VEEVRREA---------------FSDGNIEVKLC---
Mmu.3/1-433 IVR-----ETFSPGQKPGNSGISMMQKRAKLSRSQRP-VAEAKGDL---------------FSDANVDVKLCEQ-
Hsa.4/1-431 IVN-----KTFSPAQRHGNSGITMMRKKAKFSLRENP-VEETKGEA---------------FSDGNIEVKLCEQ-
Dre.13/1-422 IRR---SSHRVDVKDKSKVLFCKSARQDEEISVMPRIHIIDQVQYA---------------RSNMRTSMSFLEE-
Ame_871850/1-412 LRR----PRRLDGAPERANLSVQFSRPLKRQAF----------------------------MGNDSANMDQNGG-
Dre.12/1-426 LRR---PVQQSVPRQNLSVHFSKHLKTEAFSGAEEHLEPQHNRLLS---------------SSQGELEISTAFP-
Tni.2/1-441 VRK----PNQQVAAVAAPKLSVHFIKPQSREAFVEADQARNLQENS----------------AERGPASSSHGH-
Tru.59/1-439 IRK----PNQQAAAAVAPKVSVQFIKPQSREAFVEADEGQSQNQSS----------------AEEGPACSSHGR-
Gac.V/1-440 LRK----PNLQRGAAVVPKASVQFIKPRSREAFPESGEGDGANQLS----------------AEKGPSTSLQGK-
Ola.19/1-439 IRK----SNQQRGATVAPRLSVQFIKPQSREAFLNPDEGNSSEQAS----------------AEKGASSSLHRE-
Aja_sc551/1-426 LHK----PIQRPIPMERPKLTVHFTRPAKEDAFLHNTPPVHGSGTA---------------PSGTSHREAFTAF-
Loc_211_5/1-437 VRR-----RNPSRQRAVSAERPKPNVLFTNATRRGRFQGGTPTQGI--------------QFNLSEEAVSASSHA
Loc_LG1/1-359 ----------PRPHRTHPTSH--
Lch.126583/1-421 FKR------SRHGENRVGQEQAT-GRPNIGPSRLLKKKDKRNVQSH---------------LSSENLELKLFDQV
Psi.207659/1-320 RLR--------SPEPADKLESQQLERARMGPSNFGVRKEGRSLHSH---------------LSAENLELRLCEQV
Xtr.172779/1-431 HFR-----AQEPVEPADKRDSQQSERPKGGTTMFFARRDGRSGQSH---------------LSAENLELRLCEQF
Pma.476500/1-352 LMC------------RRP---
Lch.JH126808/1-393 RKK--------RSNMQIGVSPVDSANPRNEAQQSSVGHQRVSIMHL----EPFQVWEPNTTQCNSFPPVHLF---
Dre.21/2-456 NSC---SCWSWRSHKRVGISTVDSNEPRQGLETTYRQRGIRSWSKPAWE------AEPERPTSNTFPAMNLLSAI
Ame_872338/1-458 FLG---SAHRWKGRSRVG---ISSSEPRTATERTERRPGLRPWSKVAWEAEPNGTAEPDVPASHTFPNINLFSAI
Aja_QRFPR3/1-321 RGKGWCPPRQLRWKVRAGVSANEPPCLRKCVERDTNR-GARQWSRV--------AWEAEPTTSRTFPQSHLFRV-
Loc_73_1/1-434 RGRPRWLAGRLGDRARVGVSAIDSLHLRDRSEHSLLGAGAHQWSRV--------AWESELHTLNPFPQVHLFQG-
Conservation
000------000000203000000000000000000000-00000000---------------0000000000000-
Quality
Consensus

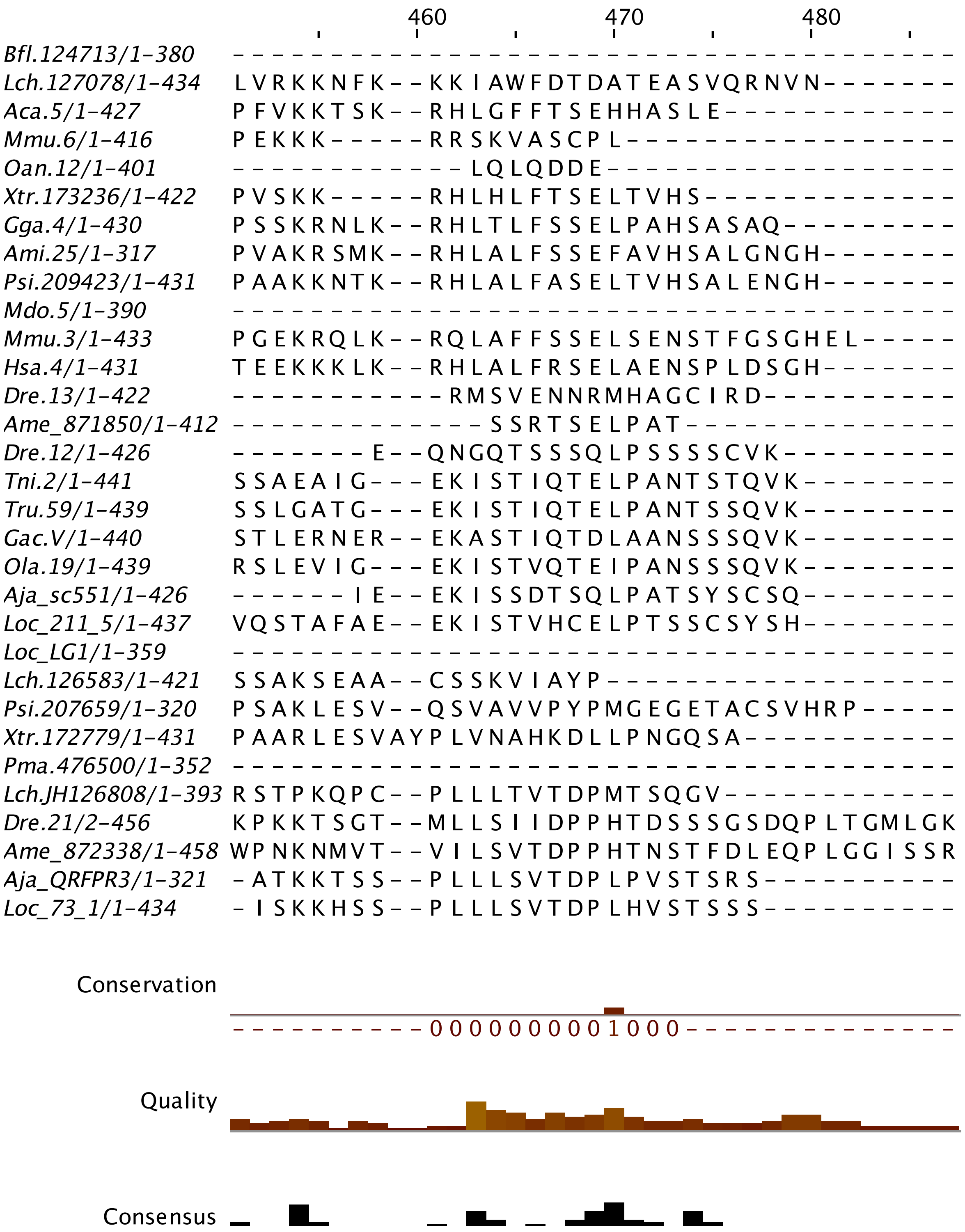
460
470
480
Bfl.124713/1-380 -------------------------------------
Lch.127078/1-434 LVRKKNFK--KKIAWFDTDATEASVQRNVN-------
Aca.5/1-427 PFVKKTSK--RHLGFFTSEHHASLE------------
Mmu.6/1-416 PEKKK-----RRSKVASCPL-----------------
Oan.12/1-401 ------------LQLQDDE------------------
Xtr.173236/1-422 PVSKK-----RHLHLFTSELTVHS-------------
Gga.4/1-430 PSSKRNLK--RHLTLFSSELPAHSASAQ---------
Ami.25/1-317 PVAKRSMK--RHLALFSSEFAVHSALGNGH-------
Psi.209423/1-431 PAAKKNTK--RHLALFASELTVHSALENGH-------
Mdo.5/1-390 -------------------------------------
Mmu.3/1-433 PGEKRQLK--RQLAFFSSELSENSTFGSGHEL-----
Hsa.4/1-431 TEEKKKLK--RHLALFRSELAENSPLDSGH-------
Dre.13/1-422 -----------RMSVENNRMHAGCIRD----------
Ame_871850/1-412 -------------SSRTSELPAT--------------
Dre.12/1-426 -------E--QNGQTSSSQLPSSSSCVK---------
Tni.2/1-441 SSAEAIG---EKISTIQTELPANTSTQVK--------
Tru.59/1-439 SSLGATG---EKISTIQTELPANTSSQVK--------
Gac.V/1-440 STLERNER--EKASTIQTDLAANSSSQVK--------
Ola.19/1-439 RSLEVIG---EKISTVQTEIPANSSSQVK--------
Aja_sc551/1-426 ------IE--EKISSDTSQLPATSYSCSQ--------
Loc_211_5/1-437 VQSTAFAE--EKISTVHCELPTSSCSYSH--------
Loc_LG1/1-359 -------------------------------------
Lch.126583/1-421 SSAKSEAA--CSSKVIAYP------------------
Psi.207659/1-320 PSAKLESV--QSVAVVPYPMGEGETACSVHRP-----
Xtr.172779/1-431 PAARLESVAYPLVNAHKDLLPNGQSA-----------
Pma.476500/1-352 -------------------------------------
Lch.JH126808/1-393 RSTPKQPC--PLLLTVTDPMTSQGV------------
Dre.21/2-456 KPKKTSGT--MLLSIIDPPHTDSSSGSDQPLTGMLGK
Ame_872338/1-458 WPNKNMVT--VILSVTDPPHTNSTFDLEQPLGGISSR
Aja_QRFPR3/1-321 -ATKKTSS--PLLLSVTDPLPVSTSRS----------
Loc_73_1/1-434 -ISKKHSS--PLLLSVTDPLHVSTSSS----------
Conservation
----------0000000001000---------------
Quality
Consensus
